# The Physiological and Biochemical Mechanisms Bioprimed by Spermosphere Microorganisms on *Ormosia henryi* Seeds

**DOI:** 10.3390/microorganisms13071598

**Published:** 2025-07-07

**Authors:** Meng Ge, Xiaoli Wei, Yongming Fan, Yan Wu, Mei Fan, Xueqing Tian

**Affiliations:** 1 School of Human Settlements, North China University of Water Resources and Electric Power, Zhengzhou 450046, China; gemeng@ncwu.edu.cn (M.G.); fanyongming@ncwu.edu.cn (Y.F.); wuyan1@ncwu.edu.cn (Y.W.); 2 College of Forestry, Guizhou University, Guiyang 550025, China; 18286455347@163.com (M.F.); t1249386@163.com (X.T.); 3 Institute for Forest Resources and Environment of Guizhou, Guizhou University, Guiyang 550025, China

**Keywords:** biopriming, functional strains, germination, mechanism, physiological indicators, seed coat, seed vigor

## Abstract

The hard-seed coat of *Ormosia henryi* significantly impedes germination efficiency in massive propagation, while conventional physical dormancy-breaking methods often result in compromised seed vigor, asynchronous seedling emergence, and diminished stress tolerance. Seed biopriming, an innovative technique involving the inoculation of beneficial microorganisms onto seed surfaces or into germination substrates, enhances germination kinetics and emergence uniformity through microbial metabolic functions and synergistic interactions with seed exudates. Notably, spermosphere-derived functional bacteria isolated from native spermosphere soil demonstrate superior colonization capacity and sustained bioactivity. This investigation employed selective inoculation of these indigenous functional strains to systematically analyze dynamic changes in endogenous phytohormones, enzymatic activities, and storage substances during critical germination phases, thereby elucidating the physiological mechanisms underlying biopriming-enhanced germination. The experimental results demonstrated significant improvements in germination parameters through biopriming. Inoculation with the *Bacillus* sp. strain achieved a peak germination rate (76.19%), representing a 16.19% increase over the control (*p* < 0.05). The biopriming treatment effectively improved the seed vigor, broke the impermeability of the seed coat, accelerated the germination speed, and positively regulated physiological indicators, especially amylase activity and the ratio of gibberellic acid to abscisic acid. This study establishes a theoretical framework for microbial chemotaxis and rhizocompetence in seed priming applications while providing an eco-technological solution for overcoming germination constraints in *O. henryi* cultivation. The optimized biopriming protocol addresses both low germination rates and post-germination growth limitations, providing technical support for the seedling cultivation of *O. henryi*.

## 1. Introduction

**Figure 1:**
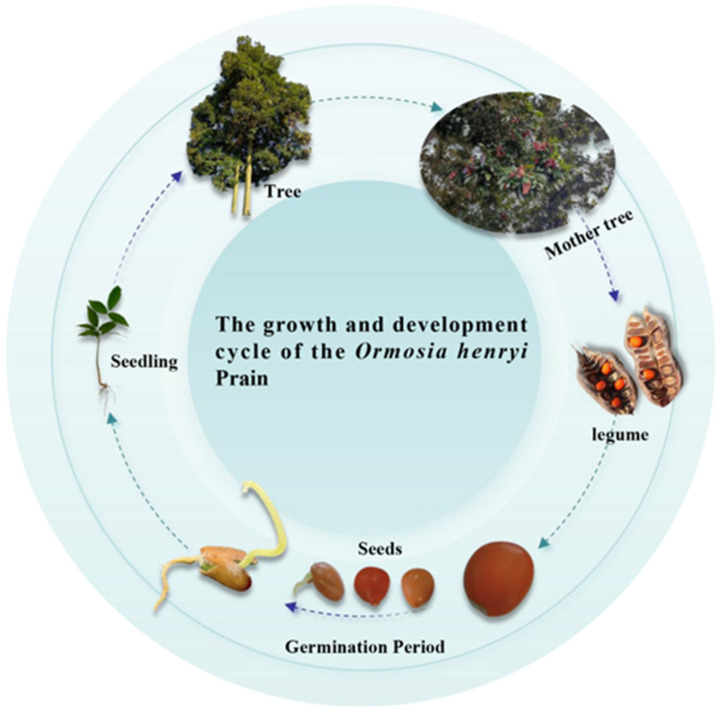
The growth and development cycle of the *Ormosia henryi* Prain.

*Ormosia henryi* Prain (Figure 1), as a species of the *Ormosia* genus of Leguminosae in China, has high ecological and economic value, and is prized as both precious timber and an ornamental landscape tree. *Ormosia henryi* seeds are pod-type seeds. Each pod contains approximately four to eight seeds, exhibiting a distinct masting phenomenon [1]. Their 1000-seed weight ranges from approximately 350 to 450 g, and seeds measure 0.75–1.27 cm in length and 0.72–1.04 cm in width. Due to their large size and weight, the seeds are primarily dispersed near the parent tree, relying on natural germination for regeneration [2]. A single *O. henryi* tree produces around 2000 pods, with a seed extraction rate of approximately 49%, indicating low seed set and utilization efficiency. Under natural conditions, the seed germination rate and seedling regeneration exhibit significant variability [3]. Furthermore, artificially cultivated seedlings demonstrate poor stress tolerance, reduced root nodulation, and drought sensitivity, resulting in weak, spindly growth and low survival rates.

In sowing practices, the seed dormancy of *O. henryi* is broken through scarification using either 80 °C hot water or 95% concentrated sulfuric acid. However, these methods often cause physical damage and nutrient loss, resulting in reduced seed vigor, compromised seedling development, and diminished stress resistance [4]. While seed germination is a critical phase in plant propagation, the hard-seeded characteristics of *O. henryi*, including impermeable seed coats and structural barriers, inherently limit germination rates and hinder regeneration. Therefore, there is an urgent need to find a suitable means to solve the problems of a low germination rate, as well as the vitality and poor stress resistance of *O. henryi* seeds, which are of great significance for expanding the germplasm resources of *O. henryi* and improving the seedling system.

**Figure 2:**
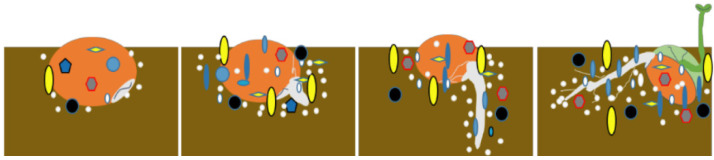
Spermosphere microorganisms at different germination stages.

The spermosphere (Figure 2), scientifically defined as the 1~10 mm microbial hotspot surrounding germinating seeds, represents a critical ecological niche where seed-derived metabolites stimulate enhanced biological activity during germination [5]. This dynamic interface hosts complex microbial communities whose metabolic interactions are fundamentally shaped by rhizodeposition processes and cross-kingdom signaling exchanges, ultimately exerting profound impacts on plant developmental trajectories [6]. Germinating seeds release exudates within the spermosphere, creating a suitable environment for microbial growth [7]. Similarly, germinating seeds recruit specific microbes into the spermosphere based on changes in these exudates [8,9,10]. Seed exudates constitute a significant component of the spermosphere alongside its microbial community, influencing the growth and development of surrounding soil microorganisms. Sugars serve as crucial energy sources for both plants and soil microbes, while amino acids play vital roles in microbial colonization, biomass accumulation, phosphate solubilization activity, and biofilm formation. Amino acids also act as mediators in plant–bacteria interactions, as many bacterial species possess genes enabling them to sense specific amino acids, thereby inducing chemotaxis in beneficial microbial communities. For instance, Dong et al. [11] reported that amino acids such as arginine, alanine, and lysine in cotton root exudates significantly promote the aggregation of the *Bacillus subtilis* strain NCD−2. Increased abundance and the colonization intensity of NCD−2 correlate with enhanced biocontrol efficacy against cotton Verticillium wilt. Consequently, the interactions between seed exudates and spermosphere microorganisms significantly impact seed germination and the recruitment of beneficial microbes within the spermosphere [6].

Contemporary research has elucidated the functional significance of specific spermosphere microbiota in modulating seed physiology across major agricultural systems: *Bacillus velezensis* enhances maize (*Zea mays*) germination through dual mechanisms of antifungal metabolite production and phytohormone regulation [12]. *Methylobacterium oryzae* orchestrates tomato (*Solanum lycopersicum*) seed activation via ACC deaminase-mediated ethylene modulation and auxin biosynthesis [13]. *Sphingomonas melonis* demonstrates rice (*Oryza sativa*) spermosphere colonization efficiency through biofilm-mediated pathogen exclusion and ROS-scavenging enzymatic systems [14]. Notably, diverse microbial taxa including *Pseudomonas* spp. [15], Bacteroidetes strains [16], and *Azospirillum brasilense* [17] have been shown to exhibit growth-promoting effects through nitrogen fixation, nutrient solubilization, and stress tolerance enhancement. This accumulating evidence strongly supports the paradigm that spermosphere microbiota serve as biochemical catalysts initiating critical germination processes. Despite these advances in herbaceous species, significant knowledge gaps persist regarding the microbial drivers of germination in perennial woody plants.

Seed germination, constituting a pivotal yet vulnerable phase in plant ontogeny, represents a metabolically intensive process susceptible to environmental perturbations and biotic challenges. To address the instability of this developmental transition, the biopriming method has been developed as an ecologically sustainable approach for synchronizing germination schedules while enhancing stress resilience [18]. Seed priming is a pre-sowing seed treatment technique that prepares the plant for future adversity by pre-exposing it to a known initiator, leading to greater survival under adverse environmental conditions. The seed priming technique involves two principal modalities: biotic and abiotic priming; the technique includes beneficial microorganisms that are inoculated the surface of a seed to establish antagonistic effects and achieve a promotion of the germination rate, seed vigor, and emergence uniformity, which is often called “biopriming” [19].

Empirical evidence has demonstrated the biostimulatory potential of a spermosphere microbial community. Rehman A, et al. [20] found that wheat yield was improved by the method of seed priming through a combination of microorganisms and Zn. Cyanobacterial inoculants enhanced maize (*Zea mays*) germination indices through the dual modulation of nitrogen metabolism and micronutrient bioavailability [21]. *Pseudomonas fluorescens* elevates East Indian sandalwood seeds’ germination efficiency via synergistic production of the plant growth hormone, organic acid, a fixation of atmospheric nitrogen, the solubilization of phosphate, and antibiotics production [18]. These findings underscore the potential for spermosphere microorganisms to reprogram seed metabolism through reactive oxygen homeostasis modulation, reserve mobilization enzyme activation, and plant growth hormone production.

Based on the above studies, whether the inoculated indigenous or non-indigenous spermosphere strains played an important role in seed germination still needs to be determined. Previous research has confirmed that spermosphere bacteria promote the germination of *O. henryi* seeds by regulating seed metabolic pathways [22]. Furthermore, by investigating the differences in spermosphere microbial communities across various soil media, the dynamics of spermosphere bacterial communities during different germination stages, functional predictions, and changes in the spermosphere bacterial network structure over germination time, we elucidated key bacterial species promoting *O. henryi* seed germination in natural soil. It was also revealed that the spermosphere bacterial community exhibits a functional group transition pattern across germination stages: shifting from decomposer-dominant, to antagonist-dominant, and finally to nutrient provider-dominant communities [3]. These findings demonstrate that spermosphere bacteria collectively regulate the natural germination of *O. henryi* seeds through synergistic interactions. However, the specific physiological and biochemical mechanisms by which individual bacteria promote germination remain poorly understood.

Therefore, this study focuses on hard-seeded *O. henryi*, using functionally superior spermosphere bacterial strains isolated and screened from wild *O. henryi* spermosphere soil as inoculants. The aims of this study are (1) to characterize and identify the functional traits and molecular taxonomy of the spermosphere bacterial isolates, (2) to investigate microscopic structural changes in the seed coat induced by spermosphere bacteria during the early germination phase, (3) to determine the differences in hormone levels, storage compounds, energy status, and seed germination-related enzyme activities between bioprimed seeds and sterilely-primed seeds at various germination stages, and (4) to elucidate the internal physiological and biochemical changes triggered by spermosphere bacterial priming, thereby revealing the underlying mechanisms driving *O. henryi* seed germination. This research will provide a theoretical basis for the chemotaxis and long-term colonization of functional spermosphere bacteria, ultimately aiming to develop these beneficial strains into green bio-fertilizers for practical application.

## 2. Materials and Methods

### 2.1. Isolation and Purification of Spermosphere Strains

The spermosphere soils of the two functional strains isolated and purified were, respectively, from Pingtang County and Guanling Buyi and Miao Autonomous County in Guizhou Province, China. Spermosphere soil suspension was prepared by homogenizing 10 g of soil with 90 mL of sterile water and six glass beads in a 250 mL conical flask. The mixture was agitated at 180 rpm for 30 min using a magnetic stirrer. Serial dilutions (10^−3^, 10^−4^, 10^−5^, 10^−6^, 10^−7^) of the suspension were prepared. Aliquots (0.1 mL) from each dilution were spread-plated onto a beef extract peptone agar medium. After incubation at 28 °C for 2–4 days, morphologically distinct colonies were aseptically subcultured onto fresh beef extract peptone agar plates using an inoculating loop. Pure strains were obtained through iterative streak-plate isolation until stable colony phenotypes were observed.

### 2.2. Determination of Functional Activity of Spermosphere Strains

The purified strains were inoculated on solid mediums [23] for amylase production, cellulase production, phosphate-solubilizing, potassium-solubilizing, and nitrogen-fixing, respectively. The inoculated strains were cultured at 30 °C for 5 days, with three biological replicates per treatment. The capacities of starch hydrolysis, cellulose hydrolysis, phosphate-solubilization, nitrogen-fixation, and potassium-solubilization were quantified by calculating the ratios of functional zone diameters to the colony diameters.

### 2.3. Molecular Identification of Spermosphere Strains

Genomic DNA was extracted from purified functional bacterial strains using a Bacterial Genomic DNA Extraction Kit (Sangon Biotech Co., Ltd., Shanghai, China). DNA samples (1–2 μL) were electrophoresed on 1% agarose gels containing GelRed at 110 V for 30 min, with the DL2000 DNA Marker as a reference. All samples exhibited clear, singular high-molecular-weight bands without degradation or low-molecular-weight smearing. The 16S rRNA gene was amplified by PCR using the universal primers 27F and 1492R [24] (5′-GGTTACCTTGTTACGACTT-3′), and the PCR products were purified by the 2× BenchTop™ Taq Master Mix (BIOMIGA, San Diego, CA, USA). The 50 μL reaction mixture contained the following: 25 μL of Master Mix, 2 μL of template DNA, 2 μL of each primer, and 19 μL of ddH_2_O. The procedure conditions of PCR amplification were as follows: pre-denaturation (1 min 30 s at 94 °C), denaturation (30 s at 94 °C), annealing (30 s at 57 °C), extension (60 s at 72 °C), and final elongation (5 min at 72 °C). The final PCR amplified products were stored at 4 °C. Among them, the steps of denaturation, annealing, and extension were continuously cycled 30 times. Purified PCR products underwent bidirectional Sanger sequencing using the dideoxy chain termination method (ABI 3730 xl, Sangon Biotech, Shanghai, China). The sequences were blasted in the GenBank database to obtain accession numbers. Phylogenetic trees were constructed using MEGA 7.0 to determine taxonomic classification.

### 2.4. Biopriming Experimental Design

The seeds used for the biopriming experiment were collected in late November 2021 from the single mother tree of *O. henryi* in Qinglong County (105°13′23.07″ W, 26°0′53.97″ N), Guizhou Province, China, with a 1000-grain weight of 362.35 ± 8.38 g. The surface dirt was cleaned with sterile water, and healthy, full, and uniform size seeds were selected for the test. The two strains (T1 and T2) were isolated and purified from the soil and inoculated onto *O. henryi* seeds to assess physiological indices and germination rates. The strains were transferred into a beef extract–peptone liquid medium [23] and cultured overnight at 30 °C with continuous shaking at 150 rpm. The bacterial suspension was adjusted to an OD_600_ of 1.0 (about 10^8^ CFU·mL^−1^).

The seeds were soaked in 4% sodium hypochlorite (NaClO) and 75% ethanol for 10 min and 30 s, respectively, and rinsed 2–3 times with sterile water for surface sterilization. The surface-sterilized seeds were then immersed in respective bacterial suspensions (different strains) for 4 h under controlled conditions. Following surface sterilization, the seeds were transferred to germination chambers lined with pre-saturated sterilized absorbent cotton (pre-treated with sterilized distilled water). The control groups included (1) seeds were soaked in sterilized water (CK1), and (2) seeds immersed in a sterilized beef extract–peptone liquid medium (CK2). Each chamber contained 70 seeds, with six biological replicates arranged in a split-plot design—with three replicates allocated for destructive sampling, and the other three replicates reserved for germination rate quantification and physiological index measurements. The germination conditions were maintained at 25 °C with a 12 h photoperiod during daytime, and 20 °C with a 12 h dark period at night. All treatments were inoculated twice during the experiment, with 10-day intervals between inoculations. Throughout the experimental period, the seeds received regular quantitative water supplementation.

Germination was defined as the period spanning from initial seed imbibition to epicotyl extension. Samples were collected at four developmental stages: (I) imbibition, (II) radicle protrusion, (III) radicle elongation, and (IV) epicotyl extension. All collected samples were processed uniformly for subsequent analysis.

### 2.5. Determination of Germination Indicators

The germination rate (GR) was counted after 20 days, and the germination index (GI) and germination potential (GP) were calculated through the following formulas.

GR = (seed germination/number of tested seeds) × 100%(1)

GP = number of germinated seeds at germination peak/number of tested seeds × 100%(2)

In Formula (2), the percentage of the number of seeds germinated within the first 1/3 period (7 d) specified in the germination test to the number of seeds tested is generally taken as the standard.

GI = ∑Gt/Dt(3)

In Formula (3) [25], Gt represents the number of germination seeds on day t; Dt represents the number of days of germination. Take the arithmetic mean of the parallel measurement results as the final analysis result, and retain the calculation result to two decimal places.

### 2.6. Observation of Seed Coat Microstructure

Samples of the seed coats (inoculated and uninoculated with the strain of T1) from the same part in the discoloration stage were taken. The imbibition stage of seeds was respectively selected, then the seeds were fixed with an FAA fixative solution (48 h), and pumped. The preparation method and steps of paraffin sections were as follows: The fixed seed coats were dehydrated in a series of concentrations of ethanol 75%, 85%, 90%, 95%, 100%, and 100% for 4 h, 2 h, 2 h, 1 h, 30 min, and 30 min, respectively. Then, they were placed in a room temperature setting to obtain transparency, with ratios of ethanol to xylene of 1:1, 0:1, 0:1, and 0:1, respectively, and the transparency time was 30 min for each. After that, the xylene in the tissue was slowly replaced with xylene and paraffin in a solution with volume ratios of 1:1, 0:1, and 0:1 at 60 °C~62 °C, and each concentration was treated for 3 h. After the waxing was completed, the tissue was embedded and trimmed, and cut into 8 μm thick sections in a paraffin sectioning machine (Laica RM2235, Wetzlar, Germany). After pasting, spreading, and baking, the dried sections were immersed in xylene for dewaxing for 40 min, and rehydrated in ethanol series concentration gradients (100%, 100%, 75%) for 5 min. After rehydration, the sections were stained with 1% toluidine blue for 2–5 min, immersed in tap water for 30 s, then 95% ethanol for about 20 s, and finally, dehydrated with anhydrous ethanol 2 times, for 5 min each; they were also made transparent with xylene after being exposed 2 times, for 5 min each. The transparent sections were permanently sealed with neutral gum and coverslips. Finally, the sections were observed under an optical microscope (Laica DM2500, Wetzlar, Germany) and typical images were selected for photographing.

### 2.7. Determination of Physiological and Biochemical Indexes of Seeds

Germinating seed samples were collected at four stages: imbibition (I), radicle protrusion (II), radicle elongation (III), and epicotyl extension (IV). All samples (including the seed coat and cotyledon) were ground with liquid nitrogen and mortar, and sieved through 20 mesh to determine physiological and biochemical indexes. All samples were collected and stored at −80 °C, and finally determined uniformly.

Gibberellin and abscisic acid levels were determined by an enzyme-linked immunosorbent kit (ELISA) (Jiangsu Meimian Industrial Co., Ltd., Yancheng, China); glucose content was determined by the hexokinase method (HK). Soluble protein content was determined by the BCA (bicinchoninic acid) method, the cellulose content was determined by sulfuric acid-anthrone colorimetry, and amylase activity was determined by the 3,5-dinitrosalicylic acid method. All physiological and biochemical activity indicators except endogenous hormones were determined using the kits of Jiangsu Grace Biotechnology (Suzhou, China) [26].

### 2.8. Statistical Analysis

Excel table and SPSS 26.0 were used for data processing. Mean ± SE was used for seed germination indicators and physiological and biochemical indexes at different stages of seed development after inoculation with a strain. Prior to performing one-way ANOVA, data should undergo normality testing and a homogeneity of variance assessment. If the normality assumption is violated, apply logarithmic transformation to approximate normal distribution. When homogeneity of variance is confirmed, use LSD post hoc testing following ANOVA. If heterogeneity of variance is detected, employ Dunnett’s T3 nonparametric analysis. Two-way ANOVA was used to study the primary effects of the germination stages and biopriming treatments and their interactions on various physiological and biochemical indexes. The Pearson correlation coefficient was used to estimate the correlation between germination indexes and physiological and biochemical indexes. A principal component analysis (PCA) was employed to further analyze the physiological and biochemical indicators of *O. henryi* seeds in different germination stages. A difference was considered statistically significant at the probability level of *p* < 0.05. Figures were produced using OriginPro Version 2024 (OriginLab Co., Northampton, MA, USA).

## 3. Results

### 3.1. Functional Characteristics of Spermosphere Strains

The two strains isolated from the spermosphere soil of *O. henryi* had multiple functional properties (Table 1). The strain from the T1 treatment not only produced amylase and cellulase, but also had the effects of organic phosphorus-solubilization, nitrogen fixation, and potassium solubilization. However, it had no effect on inorganic phosphorus-solubilization. The strain from the T2 treatment had the effect of phosphorus-solubilization and nitrogen fixation, but no role of amylase and cellulase production was found. The two strains may promote the seed germination and seedling growth of *O. henryi*.

### 3.2. Effects of Spermosphere Strains on Seed Germination Indexes

The germination rate, germination index, and germination potential of *O. henryi* seeds treated with different priming treatments (Table 2) were significantly different. The germination rate, germination index, and germination potential of T1 were the highest, and its germination rate was 76.19%, which was 14.76% and 16.19% higher than that of CK1 and CK2, respectively. The germination index and germination potential were 103.02 and 50.95%, respectively, indicating strain T1 had the best germination effect, followed by strain T2, which exhibited germination rate, germination index, and germination potential values of 73.81%, 92.37, and 45.24%, respectively. The germination rate of strain T2 was 11.75%, which was 13.18% higher than that of CK1 and CK2, respectively. However, there was no significant difference between T1 and T2, indicating that both T1 and T2 strains significantly increased the germination rate of *O. henryi* seeds.

### 3.3. Effect of Bacterial Inoculation on the Microstructure of Seed Coats

The emergence of the radicle through the hardened micropyle (the germination aperture) represents a critical developmental transition. It is during this transition that the seed coat loses its function as a germination barrier, which underscores the need for a comprehensive investigation of a seed coat’s microstructural characteristics during the early germination stages. Future studies should mainly focus on elucidating the enhancement effects of biopriming on seed germination physiology.

**Figure 3:**
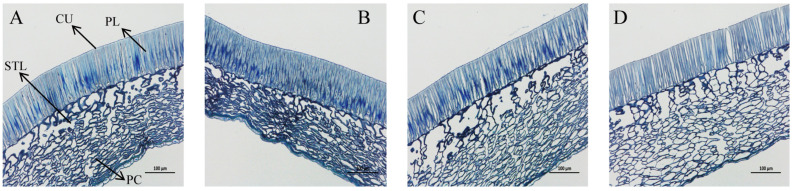
Seed coat microstructure of *O. henryi* seed at the early stage of germination. (**A**): Seed coat uninoculated with spermosphere strain at discoloration stage (BPM); (**B**): seed coat inoculated with T1 strain at discoloration stage (BPM + T1). (**C**): Seed coat uninoculated with spermosphere strain at imbibition stage (BPM); (**D**): seed coat inoculated with T1 strain at imbibition stage (BPM + T1). BPM: beef extract–peptone liquid medium; CU: cuticle; PL: palisade tissue layer; STL: sclerenchymatous cell; PC: parenchyma cell.

During early imbibition, both the T1-inoculated and non-inoculated seeds showed no observable cuticle ruptures (Figure 3A,B), with tightly packed Sclerenchymatous cells and parenchyma cells. Whereas, after imbibition, distinct structural modifications were observed during the imbibition germination stages (Figure 3D): firstly, a pronounced stomatal formation was found in the palisade tissues, indicating a rupture of seed coat integrity, and secondly, an expansion of the intercellular spaces in parenchyma cells was observed. These observations demonstrate that an inoculation of the spermosphere bacterial strain may accelerate seed permeability through its own functional characteristics, thereby overcoming permeability barriers more efficiently.

### 3.4. Effects of Spermosphere Strains on Endogenous Hormones in Seeds

**Figure 4:**
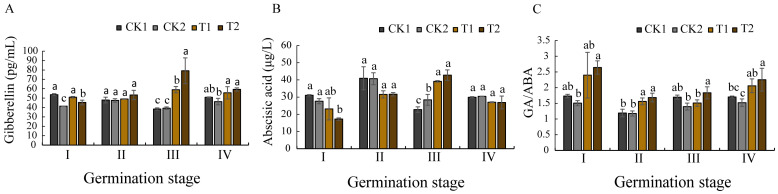
Effects of different priming treatments on endogenous hormones at different germination stages. (**A**) the gibberellin concentrations (GA), (**B**) the abscisic acid concentrations (ABA), (**C**) the ratios of GA to ABA. Lines across the data bars represent the critical levels of respective priming treatments during different germination stages. Different letters indicate significant differences between treatments of the same germination stage. Significant differences are shown at *p* < 0.05. The same below.

The endogenous hormones of *O. henryi* seeds exhibited dynamic fluctuations at different germination stages [imbibition (I), radicle protrusion (II), radicle elongation (III), and epicotyl extension (IV)] (Figure 4). Biopriming with spermosphere bacterial strains induced significant differences in endogenous hormones. Gibberellin (GA, the same as below) concentrations in the T1- and T2-treated seeds were significantly higher than those in the control groups (CK1 and CK2) during stages III and IV, exhibiting a characteristic biphasic pattern (an initial increase peaking at stage III followed by a decline) (Figure 4A). The dynamic changes in the abscisic acid (ABA, the same below) levels in the biopriming-treated groups were similar to those of GA, though their lowest concentrations occurred at stage I (Figure 4B). The results suggested that biopriming significantly suppressed ABA biosynthesis during the germination initiation phases (stage I), and markedly enhanced GA production during the active growth phases (stage III). The calculated ratio of GA to ABA (GA/ABA, the same as below) revealed elevated values in the treated groups at stage I compared to the subsequent stages (Figure 4C), indicating that biopriming regulated hormones homeostasis during early germination, thereby establishing a low-ABA microenvironment conducive to seed germination.

### 3.5. Effects of Spermosphere Strainson Storage Substances in Seeds

**Figure 5:**
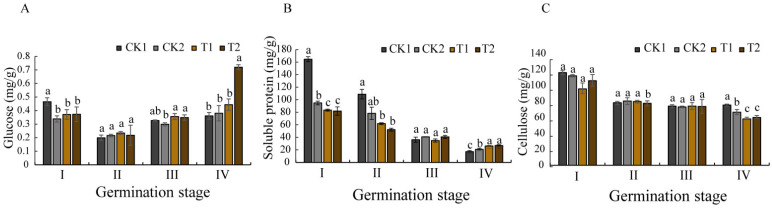
Effects of different priming treatments on stored substances and cellulose in different germination stages. (**A**) the glucose contents, (**B**) the soluble protein contents, (**C**) the cellulose contents.

An analysis of the stored substances during *O. henryi* seed germination revealed changes in the different stages under biopriming treatment (Figure 5). Biopriming eventually induced a significant elevation in glucose accumulation compared to the control groups (Figure 5A). While soluble protein content exhibited a gradual decline across the germination stages (Figure 5B), the bioprimed seeds maintained significantly higher concentrations than CK1/CK2 at stage IV (*p* < 0.05). Cellulose content demonstrated a gradual decrease, with the biopriming-treated seeds showing obviously lower levels than the controls at germination stages I and IV (Figure 5C).

These shifts of stored substances suggest that biopriming enhances energy provision through elevated glucose availability, and sustains nitrogen reserves via delayed protein catabolism. Cellulose is the main component of plant cell walls, and the observed reduction likely reflects an enzymatic degradation of the seed coat epidermal cell walls, effectively breaking physical dormancy barriers. The increase of the GA/ABA ratio and carbohydrate mobilization caused by biopriming treatment establishes an optimized biochemical environment for germination activation and post-germinative growth in this hard species.

### 3.6. Effects of Spermosphere Strains on Amylase Activity in Seeds

**Figure 6:**
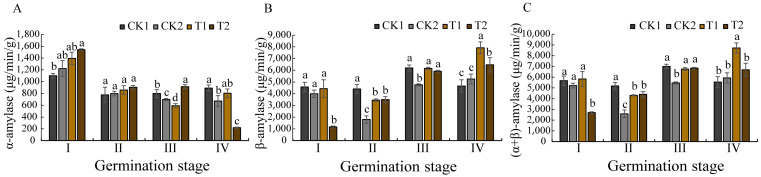
Effects of different treatments on amylase activity at different germination stages. (**A**) the α-amylase activities, (**B**) the β-amylase activities, (**C**) the (α + β) amylase activities.

Compared with the control groups, the following figure revealed a significantly different pattern of amylase activities during the germination of the *O. henryi* seeds bioprimed by spermosphere strains (Figure 4). In the inoculated T1-treated strain, α-amylase (Figure 6A) and total amylase (α + β) activities (Figure 6C) displayed a biphasic response (an initial decrease followed by a progressive increase) across the germination stages. Moreover, peak α-amylase activity occurred at stage I, while β-amylase activity reached maximum levels at stage IV (Figure 6B). By inoculating with the T2-treated strain, α-amylase exhibited a monotonic decline from stage I to IV, contrasting with the progressive increases in β-amylase and total amylase (α + β). These differential patterns demonstrate that biopriming with T1 enhances early-stage metabolic activation through α-amylase-driven starch mobilization (stage I), and the treatment of inoculation with the T2 strain preferentially sustains late-phase energy provision via β-amylase dominance (stage IV). The result suggests that the coordinated amylase regulation may correlate with accelerated seed coat rupture and improved germination efficiency, confirming that both the T1 and T2 strains can break the physiological dormancy of *O. henryi* seeds by biopriming the regulation of amylase activities.

### 3.7. Analysis of Physiological Index Parameters

**Figure 7:**
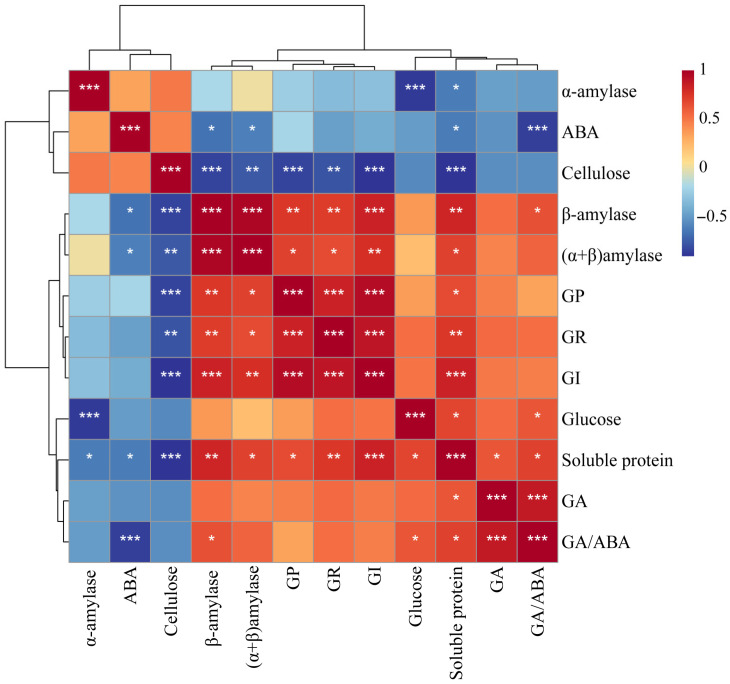
Correlation heatmap between physiological indexes and germination indicators. *, significant differences are shown at 0.01 < *p* ≤ 0.05; **, significant differences are shown at 0.001 < *p* ≤ 0.01; ***, significant differences are shown at *p* ≤ 0.001.

The two-way ANOVA can not only examine the influence of two factors on a dependent variable, but also examine the interaction between the two factors, thus affecting the continuous variable. As presented in Table 3, the analysis revealed two key findings: Firstly, the germination stage exerted statistically significant main effects (*p* < 0.05) across all the measured indexes. Priming treatment demonstrated significant main effects (*p* < 0.05) on gibberellin content, GA/ABA ratio, as well as β-amylase, total amylase, soluble protein, cellulose, and glucose levels, while showing no significant impact on abscisic acid and α-amylase. Secondly, significant interaction effects (*p* < 0.05) were observed between the two treatments for gibberellin, β-amylase, total amylase, soluble protein, cellulose, and glucose levels. Given that the germination parameters were quantified at germination stage IV, the subsequent correlation analyses focused specifically on this developmental phase (Figure 7). The results demonstrated significant positive correlations (*p* < 0.05) between three physiological indexes (β-amylase, total amylase, and soluble protein) and all the germination parameters (germination rate, germination potential, and germination index). Conversely, cellulose content showed significant negative correlations (*p* < 0.01) with these germination metrics. These findings suggest that β-amylase, total amylase, and soluble proteins may function as positive regulators of seed germination, whereas cellulose accumulation appears to exert inhibitory effects.

### 3.8. Principal Component Analysis of Physiological and Germination Parameters

**Figure 8:**
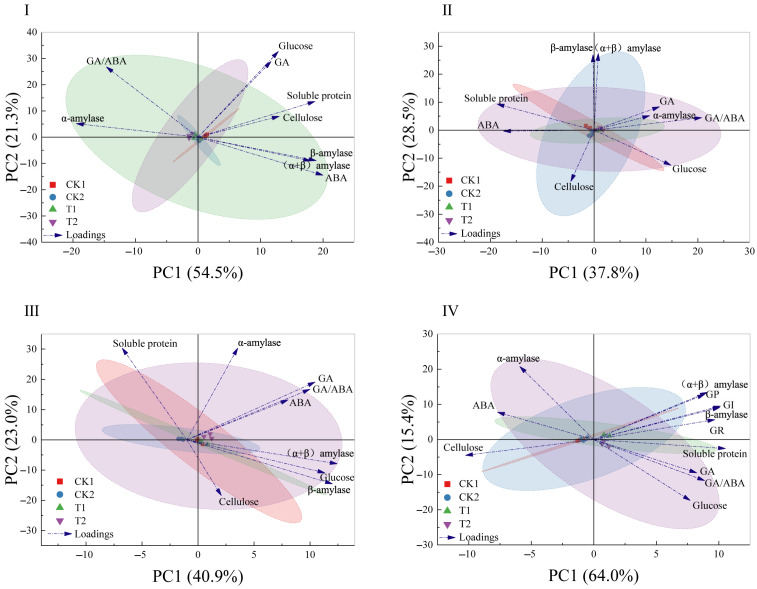
Relationship analysis of physiological and biochemical indicators in different germination stages. (**I**): Imbibition stage, (**II**): radicle protrusion stage, (**III**): radicle elongation stage, and (**IV**): epicotyl extension stage. Confidence ellipses of different colors represent different treatments, indicating the distribution of samples in the principal component space.

In the principal component analysis (PCA) of the physiological and biochemical indicators in the germination stage of imbibition (Figure 8I), the CK1 treatment was separated from the T2 treatment by the first principal component (PC1, 54.5%). Among the determinants of PC1, soluble protein content, cellulose content, β-amylase activity, (α + β) amylase activity, and ABA concentration were positively distributed, while the ratio of GA/ABA and α-amylase activity were negatively distributed. The main determinants of the second principal component (PC2) were glucose, GA, and GA/ABA.

In the PCA of the physiological and biochemical indicators in the germination stage of radicle protrusion (Figure 8II), CK1 and CK2 were separated from the T1 and T2 treatments by PC1 (37.8%). Among the determinants of PC1, the ratio of GA/ABA, GA concentration, and glucose content were positively distributed, while soluble protein content and ABA concentration were negatively distributed. The main determinants of PC2 were β-amylase, (α + β) amylase, and cellulose.

In the PCA of the physiological and biochemical indicators in the germination stage of radicle elongation (Figure 8III), CK2 was separated from the T1 and T2 treatments by PC1 (40.9%). Among the determinants of PC1, β-amylase activity, (α + β) amylase activity, glucose content, GA concentration, and the ratio of GA/ABA were positively distributed, while soluble protein content was negatively distributed. The main determinants of PC2 were soluble protein, α-amylase, and cellulose.

In the PCA of the physiological and biochemical indicators in the germination stage of epicotyl extension (Figure 8IV), CK1 and CK2 were separated from the T1 and T2 treatments by PC1 (64.0%). Among the determinants of PC1, soluble protein content, GI value, GP value, GR value, the ratio of GA/ABA, β-amylase activity, and (α + β) amylase activity were positively distributed, while cellulose content and ABA concentration were negatively distributed. The main determinants of PC2 were α-amylase, (α + β) amylase, and glucose.

Through the comparison of the principal component analysis of various physiological and biochemical indicators at different germination stages, it was found that in all germination stages, the samples treated with T1 and T2 showed a positive distribution in PC1, while the samples treated with CK1 and CK2 mostly showed a negative distribution, but the difference between the two was small. In addition, among the determinants of PC1, the ratio of GA/ABA, β-amylase, and (α + β) amylase play decisive roles in multiple germination stages (accounting for 75%) of *O. henryi*, and they are significantly negatively correlated with ABA in germination stage IV, indicating that the GA/ABA concentration, β-amylase activity, and (α + β) amylase activity induced by T1 and T2 may play key roles in relieving physiological dormancy and promoting the germination of *O. henryi* seeds. The contributions of various physiological indicators vary at different germination stages, indicating that the germination process of *O. henryi* seeds is physiologically regulated by multiple factors. Compared with conventional priming methods, the biopriming method can induce the positive regulation of seed germination by various physiological indicators, indicating that the inoculation of T1 and T2 strains may play a positive regulatory role in the germination of *O. henryi* seeds.

### 3.9. Phylogenetic Tree of Spermosphere Functional Strains

**Figure 9:**
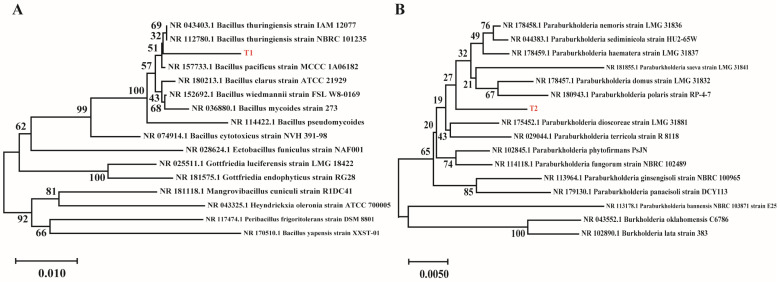
Phylogenetic tree of T1 (**A**) and T2 (**B**) strains.

The DNA sequences of the sequenced strains were submitted to the NCBI database for comparison using the Basic Local Alignment Search Tool (BLAST), and the sequences were submitted to GenBank to obtain the Accession Number for the strains T1 (Figure 9) (NCBI, Accession Number: OQ859968) and T2 (NCBI, Accession Number: OR104941). The phylogenetic tree was constructed using MEGA X 10.1.8 software with the neighbor-joining method. The results demonstrated that strain T1 exhibited the highest homology with *Bacillus thuringiensis*, while strain T2 showed the closest homology with *Paraburkholderia polaris*. Based on these findings, strain T1 was identified as belonging to *Bacillus* sp., and strain T2 was classified as *Paraburkholderia* sp.

## 4. Discussion

*O. henryi* is classified as a rare and endangered species under second-class national protection in China, and serves as a key timber species for national strategic forest reserves. This status results from its narrow, fragmented distribution, low pod yield and seed germination rates, severe anthropogenic disturbances, and limited wild populations [27]. Seed germination represents a critical prerequisite for population continuity, enabling range expansion and the colonization of new territories, while providing the foundation for maintaining genetic diversity and adaptive evolution. Given its low vegetative propagation success, *O. henryi* primarily relies on seedling propagation. However, cultivation faces significant constraints: low germination rates, poor stress tolerance in seedlings induced through physical/chemical methods, and irregular growth patterns collectively impede population regeneration and expansion.

Recent research on dormancy-breaking mechanisms reveals two primary germination barriers: the impermeable seed coat characterized by a thick cuticle, palisade and sclerenchymatous tissues, surface wax and lipid deposits, and the presence of endogenous inhibitory hormones. These structural and biochemical features collectively cause low permeability and, consequently, minimal germination in untreated seeds. As intimate partners during germination, spermosphere microorganisms play crucial roles in mediating environmental stress responses. Being a rhizobia-symbiotic legume, *O. henryi* likely benefits from spermosphere bacteria during germination. Seed priming—a key technique for enhancing vigor through rapid germination and optimized field establishment—may be revolutionized using functionally superior spermosphere bacteria as biopriming agents. Such biopriming could positively regulate germination physiology through metabolic, hormonal, and energetic pathways.

### 4.1. Effects of Spermosphere Microorganisms on Seed Germination of Ormosia henryi

In the previous research on spermosphere bacteria in *O. henryi* seed germination, it was demonstrated that during interactions between spermosphere microbiota and seed exudates, key bacterial taxa exhibit enriched abundance and functional roles in promoting hard-seed germination, including *Bacillus* sp., norank_o__Acidobacteriales, *Nitrospira* sp. [22]. Furthermore, the following critical seed exudates were identified: L-lysine, L-isoleucine, α-D-glucose, and raffinose. These bacteria likely facilitate germination by modulating hormone- and energy-related metabolic pathways through their functional traits, exemplified by the following: galactose metabolism, lysine degradation, valine, leucine, and isoleucine degradation [3]. Concurrently, they mobilize seed energy reserves, accelerating germination kinetics. In the soils supporting high germination rates, an enriched recruitment of functional spermosphere bacteria was observed, notably *Mesorhizobium* sp., *Massilia* sp., and *Burkholderia-Caballeronia-Paraburkholderia* sp., which showed significant positive correlations with germination rates. These taxa contribute to nitrogen fixation, biocontrol, and stress tolerance.

The current findings validate previous studies. Herein, the physiological mechanism of *Bacillus* sp. T1—an indigenous multifunctional strain exhibiting phosphate solubilization, potassium release, nitrogen fixation, amylase production, and cellulase production—was revealed. This bacterium primarily enhances germination by biodegrading the impermeable seed coat, modulating the ratio of GA to ABA, and balancing amylase synthesis to release physiological dormancy. This is consistent with the research results of Hu et al. [28]; in this study, *Bacillus* QM3 significantly promoted the expression of TaGA3ox-B2 in wheat seeds, thereby inducing the biosynthesis of GA and further promoting germination. This study elucidates the biochemical basis of spermosphere bacteria-mediated germination in *O. henryi.*

### 4.2. Advantages of Biopriming and Functional Characteristics of Spermosphere Strains

As an innovative approach, seed biopriming demonstrates significant potential in improving seed health through alleviating biotic and abiotic stresses, which effectively enhances seed uniformity and seedling quality [29]. This method has been proven to be particularly beneficial for functional applications in both spermosphere and rhizosphere environments. Spermosphere microorganisms, being the primary responders to seed exudates, actively participate in metabolic dynamics during germination. Their interactions with seeds provide critical insights into the microbial-mediated metabolic mechanisms that stimulate germination [22]. Effective colonization by beneficial bacteria, particularly those originating from the host’s spermosphere, ensures sustained functional impacts on plant development [30]. Notably, the spermosphere harbors numerous beneficial bacteria exhibiting multifaceted capabilities including phosphorus solubilization, potassium mobilization, enzyme synthesis, as well as the production of iron carriers and hormones. These functional attributes collectively influence seed germination and subsequent seedling development.

The two spermosphere bacterial strains investigated in this study demonstrate distinct beneficial characteristics: both exhibit phosphorus and potassium solubilization along with nitrogen fixation capabilities, while strain T1 additionally produces cellulase and amylase. This enzymatic profile proves particularly relevant for *O. henryi* seeds, whose impermeable seed coat contains palisade tissue and stone cells containing excessive cellulose and lignin. Cellulases play a crucial role in degrading these structural components [31,32], whereas amylases facilitate starch mobilization during germination, directly impacting seedling establishment [33].

Beyond these specific functions, beneficial bacteria contribute to plant health through nutrient provisioning, phytohormone synthesis, biocontrol activities, and soil structure improvement [34,35]. Molecular identification confirmed that strain T1 belongs to the *Bacillus* sp. and T2 belongs to the *Paraburkholderia* sp. *Bacillus* species, which are widely recognized for their multifunctional applications in agriculture, particularly in crop growth promotion and yield enhancement [36]. *Paraburkholderia* strains demonstrate dual efficacy in fungal pathogen biocontrol [37] and soybean yield improvement [38], while also harboring the biodegradation genes critical for soil remediation [39]. Therefore, these two spermosphere strains will have great application value in the field of hard-seed germination.

### 4.3. Effects of Biopriming on Seed Germination Indexes

Seed priming serves as a crucial pretreatment technique in plant propagation. Research demonstrates that this method effectively enhances seed vigor and improves stress resistance [40]. However, during *O. henryi* seed germination, structural damage to cellular membranes can lead to a substantial leakage of intracellular organic compounds, consequently diminishing seed vigor [3,22]. Seed vigor is typically assessed through three key parameters: germination potential (reflecting germination speed), germination rate (indicating overall viability), and germination index (quantifying both speed and uniformity). Notably, seeds with elevated germination potential exhibit stronger vigor, synchronized germination patterns, and consistent seedling emergence. In our experimental findings, the *O. henryi* seeds inoculated with two spermosphere strains significantly outperformed both control groups (CK1 and CK2) across all germination metrics. The *Bacillus*-treated seeds achieved optimal performance with the highest germination parameters indicating maximum vigor and germination efficiency. The *Paraburkholderia*-treated seeds ranked second in germination effectiveness. These results confirm the superiority of biopriming over conventional treatments, aligning with previous reports on microbial-enhanced seed germination and seedling development [37,41]. This study establishes that biopriming effectively enhances both germination vigor and efficiency for *O. henryi* seeds, providing a robust theoretical foundation and practical framework for artificial cultivation.

### 4.4. Effects of Biopriming on Seed Hormones

Hormones serve as central regulators in plant growth and developmental processes. Seeds are regulated by hormones from maturation to subsequent germination [42,43]. Particularly, the antagonistic interaction between gibberellic acid (GA) and abscisic acid (ABA) constitutes a fundamental regulatory mechanism controlling seed dormancy and germination. GA functions as a dormancy-breaking agent that stimulates germination through the transcriptional activation of hydrolase genes and the synthesis of germination-related enzymes, while simultaneously counteracting ABA effects [44,45]. Conversely, ABA maintains dormancy by inhibiting GA-mediated mobilization of seed reserves during germination [46]. Scientific research has found that the ratio of GA/ABA will produce a dynamic balance during the growth cycle of plants. Elevated cellular GA levels correlate with accelerated growth, whereas ABA predominance results in growth retardation or arrest [47]. This hormonal balance has consequently been established as a reliable indicator of germination progression.

These experimental data revealed significant hormonal modulation in the bioprimed *O. henryi* seeds. Both T1 (*Bacillus* sp.) and T2 *(Paraburkholderia* sp.) treatments induced marked GA accumulation from germination stage III on, peaking at this phase while maintaining levels substantially higher than the control groups (CK1 and CK2). Conversely, ABA concentrations in the treated seeds were significantly reduced during stages I–II, reaching minimal values at stage I. Moreover, the value of GA/ABA was significantly higher for T1 and T2 than CK1 and CK2 at germination stage I. The results showed that the biopriming method elicited a characteristic biphasic hormonal response: an initial suppression of ABA synthesis during the early germination stages (I–II), followed by progressive GA upregulation, peaking at stage III. This coordinated regulation accelerated germination efficiency, as evidenced by the enhanced germination rates and seed vigor across the germination stages. The observed effects align with the established mechanisms of microbial-mediated hormonal regulation [39], confirming that biopriming enhances germination efficiency through GA/ABA modulation.

### 4.5. Effects of Biopriming on Storage Substances and the Enzyme Activity of Seeds

Seed germination requires substantial energy and nutrient resources to support seedling establishment [48]. Glucose is a soluble sugar, which is the basis of plant metabolism and an important energy source for plants in the growth and development process. Research has demonstrated that glucose availability directly influences seed germination efficiency and early seedling vigor. Some studies have found that amylase activity in germinating seeds is positively correlated with soluble sugar content [49], techniques enhance this process by stimulating amylase production, thereby accelerating starch hydrolysis into metabolically available monosaccharides. This study revealed that biopriming induced synchronized fluctuations in glucose content and total (α + β) amylase activity, both parameters displayed decreases first and then increases, peaking at germination stage IV. This enzymatic regulation appears interconnected with phytohormonal control mechanisms, as amylase activity is known to be modulated by the antagonistic actions of gibberellins (GAs) and abscisic acid (ABA) [50,51]. Specifically, ABA accumulation suppresses GAMYB protein expression, subsequently inhibiting GA-mediated α-amylase production [52], establishing an inverse relationship between ABA levels and α-amylase activity. Consistent with this model, our biopriming treatment effectively suppressed ABA biosynthesis during the early germination phases, resulting in enhanced α-amylase activation. Notably, we observed a temporal shift in the different amylases, with α-amylase activity progressively declining while β-amylase activity increased during the later germination stages.

The physiological impacts of biopriming extended to other critical germination parameters. Soluble protein content, a key indicator of embryonic metabolic activity, exhibited stage-dependent dynamics, initially decreasing below control levels (CK1 and CK2) during stages I–II, then surpassing the control values by stage IV. Cell wall metabolism showed similar biopriming effects, with significantly reduced cellulose content in the bioprimed seeds, which was particularly evident in the T1-treated groups at stages I and IV. This cellulose degradation is likely caused by cellulase production by the inoculated *Bacillus* strain, potentially addressing the water impermeability issues associated with *O. henryi* seed coats [4]. The enzymatic softening of sclerenchymatous cell walls appears crucial for dormancy release in this species.

In conclusion, inoculation with *Bacillus* sp. strain T1 effectively alleviates both physical and embryonic dormancy of *O. henryi* seeds. Functioning as a biopriming agent, this bacterial strain coordinates multiple physiological enhancements, including modulating the endogenous phytohormone balance to enhance amylase activity, promoting storage substance mobilization, and facilitating structural modifications in seed coat architecture. These synergistic effects collectively improve germination efficiency, enhance seed vigor, and provide essential energetic reserves for post-germinative growth.

## 5. Conclusions

Spermosphere-mediated biopriming significantly enhanced germination performance in *O. henryi*. The comprehensive effect of biopriming with *Bacillus* sp. is the best, as the germination rate reached 76.19%, and it had the ability to decompose cellulose during the early germination period, hydrolyzing starch, decomposing organophosphorus, causing nitrogen fixation, and solubilizing potassium, which played an important role in breaking the dormancy of hard seeds and improving seed vigor. This functional synergy effectively overcame both physical and physiological dormancy constraints, establishing it as a sustainable solution for propagating recalcitrant legume species.

Building upon previous findings, this study selected two functionally superior bacterial strains for biopriming treatment. Through a microscopic structural analysis and physiological assessments, we elucidated how spermosphere bacteria enhance *O. henryi* seed germination. However, the following limitations remain: the current inoculation methods employed single-strain applications only on *O. henryi* seeds, mixed inoculation strategies and stage-specific bacterial consortia were not investigated, and the sustained effects on seedling development remain unverified. Future research directions will address these gaps. An experimental validation of spermosphere bacteria (single/combined strains) needs to be conducted including determining the long-term effects on seedling growth, chemotactic mobility, and colonization capacity, and an exploration of the potential symbiotic traits in *O. henryi* seedlings.

## 6. Patents

The method of promoting the germination of *Ormosia henryi* seeds by a strain of *Bacillus* named GM-12-PT is patented, with the Patent number: ZL 2023 1 0678021.8.

## Figures and Tables

**Table 1:** The functional characteristics of spermosphere bacteria.

Strain	Amylase Production	Cellulase Production	Solubilization of Organic Phosphorus	Nitrogen Fixation	Potassium Solubilization	Solubilization of Inorganic Phosphorus
T1	1.61 ± 0.08	2.55 ± 0.15	1.50 ± 0.38	1.40 ± 0.16	1.37 ± 0.03	—
T2	—	—	1.63 ± 0.04	3.44 ± 0.11	—	1.81 ± 0.12

“—“ represents negative, indicating that it does not have this function.

**Table 2:** Effects of different priming treatments on germination indexes of *O. henryi* seeds.

Treatment	GR/%	GI	GP/%
CK1	61.43 ± 5.08 b	64.91 ± 3.20 b	36.67 ± 3.75 b
CK2	60.00 ± 2.33 b	71.73 ± 4.56 b	39.05 ± 2.94 b
T1	76.19 ± 1.78 a	103.05 ± 3.25 a	50.95 ± 2.69 a
T2	73.81 ± 3.75 a	92.37 ± 8.47 a	45.24 ± 5.51 ab

GR: germination rate; GI: germination index; GP: germination potential. Different letters indicate significant differences between treatments of the same parameter. Significant differences are shown at *p* < 0.05.

**Table 3:** F-values (numbers before parentheses) and degrees of freedom (numbers in parentheses) of two-way ANOVA for the main effects of germination stages and biopriming treatments and their interactions on physiological indexes.

Variables	*F*_stage_	*F*_priming_	*F*_stage×pri_ _ming_
GA	4.094 (3) *	12.446 (3) ***	10.169 (9) ***
ABA	23.154 (3) ***	0.836 (3)	11.318 (9) ***
GA/ABA	12.355 (3) ***	13.976 (3) ***	1.346 (9)
α amylase	141.420 (3) ***	1.238 (3)	19.626 (9) ***
β amylase	114.469 (3) ***	25.870 (3) ***	19.712 (9) ***
(α + β) amylase	95.152 (3) ***	30.258 (3) ***	18.627 (9) ***
Soluble protein	680.395 (3) ***	101.570 (3) ***	54.502 (9) ***
Cellulose	154.347 (3) ***	7.268 (3) ***	3.003 (9) **
Glucose	110.944 (3) ***	18.973 (3) ***	18.458 (9) ***

*, 0.01 < *p* ≤ 0.05; **, 0.001 < *p* ≤ 0.01; ***, *p* ≤ 0.001. All those marked with *, **, *** indicate that there is a significant main effect or interaction effect among the variables.

## Data Availability

The original contributions presented in this study are included in the article. Further inquiries can be directed to the corresponding author.

## Abbreviations

The following abbreviations are used in this manuscript:

16S rDNA	16S ribosomal DNA identification
OTUs	Operational Taxonomic Units
PValue	Probability Value
CFUs	Colony forming units
ddH_2_O	Distillation-Distillation H_2_O
OD_600_	Optical density _600_
GR	Germination rate
GP	Germination potential
GI	Germination index
GA	Gibberellic acid
ABA	Abscisic acid
PC	Principal component
PCA	Principal component analysis
DNA	Deoxyribonucleic acid
BCA	Bicinchoninic acid
PCR	Polymerase Chain Reaction
